# Generation and Release of Mitochondrial-Derived Vesicles in Health, Aging and Disease

**DOI:** 10.3390/jcm9051440

**Published:** 2020-05-12

**Authors:** Anna Picca, Flora Guerra, Riccardo Calvani, Hélio José Coelho-Junior, Maurizio Bossola, Francesco Landi, Roberto Bernabei, Cecilia Bucci, Emanuele Marzetti

**Affiliations:** 1 Fondazione Policlinico Universitario “Agostino Gemelli” IRCCS, 00168 Rome, Italy; anna.picca@guest.policlinicogemelli.it (A.P.); maurizio.bossola@unicatt.it (M.B.); francesco.landi@unicatt.it (F.L.); roberto.bernabei@unicatt.it (R.B.); emanuele.marzetti@policlinicogemelli.it (E.M.); 2 Department of Biological and Environmental Sciences and Technologies, Università del Salento, 73100 Lecce, Italy; guerraflora@gmail.com; 3 Institute of Internal Medicine and Geriatrics, Università Cattolica del Sacro Cuore, 00168 Rome, Italy; coelhojunior@hotmail.com.br

**Keywords:** biomarkers, exosomes, extracellular vesicles, geroprotective interventions, mitophagy, mitochondrial damage, mitochondrial dynamics, mitochondrial-derived vesicles (MDVs), mitochondrial-lysosomal axis, neurodegeneration

## Abstract

Mitochondria are intracellular organelles involved in a myriad of activities. To safeguard their vital functions, mitochondrial quality control (MQC) systems are in place to support organelle plasticity as well as physical and functional connections with other cellular compartments. In particular, mitochondrial interactions with the endosomal compartment support the shuttle of ions and metabolites across organelles, while those with lysosomes ensure the recycling of obsolete materials. The extrusion of mitochondrial components via the generation and release of mitochondrial-derived vesicles (MDVs) has recently been described. MDV trafficking is now included among MQC pathways, possibly operating via mitochondrial–lysosomal contacts. Since mitochondrial dysfunction is acknowledged as a hallmark of aging and a major pathogenic factor of multiple age-associated conditions, the analysis of MDVs and, more generally, of extracellular vesicles (EVs) is recognized as a valuable research tool. The dissection of EV trafficking may help unravel new pathophysiological pathways of aging and diseases as well as novel biomarkers to be used in research and clinical settings. Here, we discuss (1) MQC pathways with a focus on mitophagy and MDV generation; (2) changes of MQC pathways during aging and their contribution to inflamm-aging and progeroid conditions; and (3) the relevance of MQC failure to several disorders, including neurodegenerative conditions (i.e., Parkinson’s disease, Alzheimer’s disease) and cardiovascular disease.

## 1. Introduction

Mitochondria are intracellular organelles that participate in nearly all biological processes by ensuring energy supply, iron, and calcium buffering, signaling through reactive oxygen species, steroid hormone and heme biosynthesis, and control of cell death/survival pathways [1,2]. Recent advances in mitochondrial physiology have assigned a role for these organelles in pathways that are not canonically linked with mitochondria, such as inflammation [3]. Given the multifaceted functions of mitochondria, it is not surprising that their (dys) function has attracted considerable interest in the setting of multiple conditions (e.g., aging, cancer, neurodegeneration, cardiovascular disease, metabolic disorders) [4,5,6,7,8].

In order to guarantee efficient energy provision and proper integration of intracellular signaling, mitochondria need to remain plastic and connected with other cellular compartments. Membrane contact sites and tethering molecules are essential components of inter-organelle interactions [9,10,11]. Via these structures, mitochondria establish physical and functional interactions with the endosomal compartment [12,13] and lysosomes [14,15]. While the first system supports ion and metabolite shuttling across organelles [10,16], the interaction with lysosomes is in place to ensure the recycling of obsolete materials [14,15].

The mitochondrial-lysosomal axis is recognized as the main actor in the deployment of mitochondrial quality control (MQC). The latter involves a hierarchical network of pathways that operate through the coordination of mitochondrial proteostasis, dynamics, biogenesis, and autophagy to preserve organellar homeostasis [7].

The mitochondrial unfolded protein response (UPR^mt^) is a conserved stress-responsive system essential for mitochondrial proteostasis [17]. UPR^mt^ is composed mainly of the ATPases Associated with diverse cellular Activities (AAA) p97 and the cofactor nuclear protein localization protein 4 (NPL4) [18]. Under stress conditions, several mitochondrial stress proteins, including chaperonin 10 and 60, mtDnaJ, ATP-dependent Clp protease proteolytic subunit (ClpP), and intermembrane space AAA (i-AAA) protease supercomplex subunit (Yme1), are expressed to ensure mitochondrial proteostasis [19]. Although the regulation of the UPR^mt^ is not fully understood, activating transcription factor 5 (ATF5) has been shown to orchestrate UPR^mt^ in mammals [20].

Coordinated cycles of fusion and fission control mitochondrial shape, which is essential for adequate energy provision and dilution of damage along the network [21]. Instead, mitochondrial hyper-fission is in charge of segregating severely damaged or dysfunctional organelles [21] for their eventual disposal via a specialized form of autophagy termed mitophagy [22]. Concomitantly, mitochondrial replenishment via biogenesis maintains an adequate mitochondrial pool [23].

The establishment of mitochondrial-lysosomal contact sites has recently been included among the mechanisms participating in MQC as an additional layer of quality check involving crosstalk between the two organelles and culminating in the release of extracellular vesicles (EVs) [24]. Down this alternative degradative route, mildly damaged mitochondrial components are processed and disposed of within EVs of mitochondrial origin (mitochondrial-derived vesicles, MDVs) [25]. As such, this mechanism contributes to organelle homeostasis before whole-sale mitochondrial degradation is triggered [25].

Mitophagy dysregulation or impairment in the mitochondrial-lysosomal axis instigates the intracellular accumulation of noxious materials (e.g., damaged mitochondria, misfolded proteins, lipofuscin) [26]. In the long-term, the stalling of housekeeping systems further depresses cell recycling processes, thereby impinging on cell homeostasis and tissue integrity [26].

Here, we discuss (1) canonical MQC pathways with a special focus on mitophagy and MDV generation; (2) changes of MQC pathways during aging and their contribution to inflamm-aging and progeroid conditions; and (3) the relevance of MQC failure to several disorders, including neurodegenerative conditions such as Parkinson’s disease (PD), Alzheimer’s disease (AD), and cardiovascular disease (CVD).

## 2. Mitophagy or Generation of Mitochondrial-Derived Vesicles: Easy Come Easy Go

The fine-tuning of MQC ensures mitochondrial plasticity, disposal, and replenishment to maintain a well-functioning organelle network within the cell and to meet dynamic tissue energy demands [7].

Mitochondrial fission, under the control of the guanosine triphosphatase (GTPase) dynamin-related protein 1 (DRP1), fission protein 1 (FIS1), and dynamin 2, regulates mitochondrial division and biogenesis by modulating the rate of mitochondrial DNA (mtDNA) synthesis [27,28,29,30,31,32]. The tethering and fusion of mitochondrial membranes are mediated by the outer membrane GTPases, mitofusin (MFN) 1 and MFN2, and the inner membrane GTPase, optic atrophy 1 (OPA1). Mitochondrial fusion enables the exchange of proteins, mtDNA, and metabolites between networked organelles and allows the dilution of mitochondrial damage [27]. Eventually, mitophagy is in place to remove irreversibly damaged mitochondria and mitigate organelle dysfunction [33].

Mitophagy is a multi-step process beginning with the engulfment of damaged or unnecessary mitochondria within a degradative structure named autophagosome. Autophagosomes fuse and deliver their cargo to lysosomes and form an autolysosome wherein the engulfed material is degraded [22]. A sophisticated molecular machinery orchestrates the whole process, which may proceed via serine/threonine-protein phosphatase and tensin homolog-induced kinase 1 (PINK1)/Parkin-dependent and independent pathways [5]. PINK1/Parkin-dependent mitophagy is the best-studied pathway in mammalian cells and is described here in more detail.

**Figure 1:**
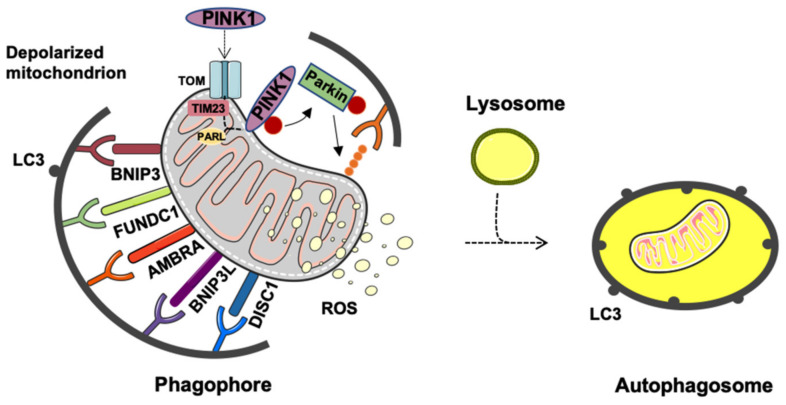
Quality control of mitochondria through mitophagy. Severely depolarized mitochondria are targeted to degradation by phosphatase and tensin homolog-induced kinase 1 (PINK1)-Parkin-dependent and independent pathways. Through the mediation of the translocase of the outer mitochondrial membrane (TOM) and the translocase of inner mitochondrial membrane 23 (TIM23), PINK1 is imported into functional mitochondria and is degraded by presenilin-associated rhomboid-like (PARL) protein. Upon mitochondrial depolarization, PINK1 accumulates at the outer mitochondrial membrane (OMM), where it recruits Parkin to trigger mitophagy. A set of OMM proteins, including FUN14 domain containing 1 (FUNDC1), autophagy and Beclin-1 regulator 1 (AMBRA1), B-cell lymphoma 2 (BCL2)-interacting protein 3 like (BNIP3L), BNIP3, and disrupted-in-schizophrenia-1 (DISC1), assists the whole process by detecting damaged mitochondria and interacting with microtubule-associated protein 1A/1B-light chain 3 (LC3). ROS, reactive oxygen species.

The proper disposal of dysfunctional mitochondria involves the coordinated activity of complex molecular machinery. In healthy, polarized mitochondria, the translocase of the outer mitochondrial membrane (TOM) and the translocase of inner mitochondrial membrane 23 (TIM23) support the import of PINK1 within the organelle followed by its cleavage by the presenilin-associated rhomboid-like (PARL) protein [34,35,36]. Conversely, the loss of mitochondrial membrane potential triggers the accumulation and stabilization of PINK1 at the outer mitochondrial membrane (OMM) [35,37]. Here, PINK1 is activated via autophosphorylation at specific serine residues [32], followed by the recruitment of Parkin from the cytoplasm to the OMM [34,38,39,40] through the phosphorylation and ubiquitination at serine 65 [41,42] (Figure 1). Once recruited, Parkin ubiquitinates proteins located at the OMM interface, such as the voltage-dependent anion channel (VDAC), Ras homolog family member T1 (RHOT1), and MFN1/2 that act as phosphoubiquitin substrates. Polyubiquitination enables the interaction of mitochondria with mitophagy adapter proteins (i.e., nuclear dot protein 52 (NDP52) and optineurin (OPTN)) and the microtubule-associated protein 1A/1B-light chain 3 (LC3) via a conserved amino acid motif (WXXL) for delivery to autophagosomes [43,44] and sequestration within the isolation membrane [45] (Figure 1). The completion of the degradative process is achieved after the accumulation of the ubiquitin-binding adaptor protein p62/sequestosome-1 on depolarized mitochondria and its binding to LC3 [45]. This event facilitates the delivery of mitochondria to autophagosomes to complete their degradation [45].

The PINK1/Parkin-independent pathway of mitophagy relies on the activity of a set of OMM-localized receptors, including B-cell lymphoma 2 (BCL2)-interacting protein 3 like (BNIP3L/NIX), FUN14 domain-containing 1 (FUNDC1), BNIP3, autophagy, and Beclin-1 regulator 1 (AMBRA1), BCL2-like 13 (BCL2L13), FKBP prolyl isomerase 8 (FKBP8), and disrupted-in-schizophrenia-1 (DISC1) [33] (Figure 1). These receptors detect damaged mitochondria by interacting with processed LC3 at phagophores via a common LC3-interacting region (LIR) motif in their cytosolic N-terminal domains. The description of these pathways does not fall within the scope of this review and can be found elsewhere [33].

The mitophagy response triggered by complete mitochondrial depolarization has been thoroughly characterized. Though, it remains unclear how the whole system makes coherent decisions on whether to keep or purge mitochondria when they are only mildly damaged. Emerging evidence shows that, at least in vitro, temporal integration of mitochondrial stress signals is mediated by the PINK1/Parkin pathway according to the magnitude and duration of mitochondrial insults [46]. While PINK1 and Parkin accumulate stably on completely depolarized mitochondria, only a transient stabilization of PINK1 is observed in response to partial mitochondrial depolarization [46]. In this context, a slow step-wise accumulation of Parkin occurs with consequent phospho-polyubiquitination and delayed mitophagy [46]. Divergent rates of mitophagy degradation may also arise from the differential phosphorylation of individual mitochondrial protein species by the activity of phosphatases and kinases [47].

The Ras-related in Brain protein 7A (RAB7A), a ubiquitously expressed small GTPase belonging to the RAB family, is a well-known regulator of mitophagy downstream of Parkin [48]. RAB7A shuttles between an active, lysosomal-localized guanosine-5’-triphosphate (GTP)-bound state and an inactive, cytosolic guanosine diphosphate (GDP)-bound state. This process is regulated by proteins with Guanine nucleotide Exchange Factor (GEF)-function, that induce GDP dissociation and GTP binding, and proteins with GTPase-Activating Protein (GAP)-function, that stimulate GTP hydrolysis [49]. Once recruited on the late endosomal-lysosomal membranes, RAB7A is activated by GEFs and interacts with effector proteins through which it regulates multiple processes involved in MQC [49]. Via these interactions, RAB7A regulates the maturation of early endosomes, the transport of intracellular material from late endosomes to lysosomes, lysosomal biogenesis, and the clustering and fusion of late endosomes and lysosomes in the perinuclear region of the cell [49]. Through its activities in endosomal trafficking, RAB7A also participates in autophagy, mitophagy, secretion of EVs [49,50], and tethering and untethering of mitochondrial-lysosomal contacts [15]. Hence, RAB7A is considered to be an effector of mitophagy involved in autophagosome biogenesis [51]. To accomplish this task, RAB7A cooperates with the Tre-2/Bub2/Cdc16 (TBC) domain family, member 15 and 17 (TBC1D15/TBC1D17) and FIS1 [51]. In particular, the interaction of TBC1D15/17 with LC3 and FIS1 is essential to coordinate RAB7A activity and guide the isolation of the pre-autophagosomal membrane that selectively engulfs damaged mitochondria [51]. The silencing of RAB7A suppresses abnormal LC3 accumulation and tubulation in TBC1D15^–/–^ cells [51]. Thus, while constitutive RAB7A activity favors the expansion of the LC3-positive isolation membrane, RAB7A inactivation may be required for the release of LC3-bound membranes from microtubules [51,52]. This confers to RAB7A further roles besides that of controlling the final step of the maturation of autophagosomes by their fusion with lysosomes [53,54]. This function seems to be mediated by the interaction of RAB7A with MFN2 during the maturation of the autophagosomal membrane [55]. Thus, RAB7A may support both autophagosome formation and maturation during mitophagy.

A recent study showed that RAB7A is involved in mitophagy also via its regulation by the retromer complex [56]. The latter is a multi-subunit complex that orchestrates the retrograde transport of materials from endosomes to the Trans-Golgi Network (TGN) or from endosomes to the plasma membrane [57]. The complex includes vacuolar protein sorting (Vps) 26, Vps29, and Vps35 that constitute a heterotrimeric cargo recognition subcomplex and sorting nexin (SNX) proteins that form hetero/homo-dimeric subcomplex of retromer. The localization of the retromer at the endosomal membrane is enabled by the simultaneous interaction of Vps35 with active RAB7A-GTP and SNX3 [49]. RAB7A activity is controlled by TBC1D5, the retromer-associated RAB7-specific GAP, that interacts with the subunit Vps29 of the retromer [58]. Due to this interaction, RAB7A localizes around damaged mitochondria and promotes their removal through Parkin-mediated mitophagy [56]. If the retromer is lost, hyperactivated RAB7A is sequestered on late endocytic membranes and cannot localize on damaged mitochondria, eventually resulting in defects in the mitophagosome formation [56].

Post-translational modifications of RAB7A also play a critical role in mitophagosome formation. RAB7A activity is regulated by the Src kinase through serine-threonine phosphorylation at serine 72 (S72) and phosphorylation of tyrosine 183 (Y183) [59]. Recently, Heo and colleagues [60] found that RAB7A S72 is a target of tumor necrosis factor receptor-associated factor nuclear factor κB (NF-κB) activator (TANK)-binding kinase 1 (TBK1), a kinase activated by the assembly of ubiquitin chains on mitochondria and by mitochondria depolarization during mitophagy. A small fraction of RAB7A is phosphorylated at S72 by TBK1 in a PINK1-Parkin dependent manner. RAB7A S72 interacts with folliculin and the folliculin-interacting protein 1 (FLCN-FNIP1) complex to recruit damaged mitochondria and promote Parkin-dependent mitophagy. Indeed, cells with mutations in RAB7A phosphorylation sites exhibit defects in the recruitment of damaged mitochondria [60].

The identification of MQC members among the regulators of mitophagy led to hypothesize the existence of inter-organelle functional connections, in particular mitochondrial–lysosomal contacts that may represent a further level of MQC [16]. The observation that defects in either of the two organelles induce impairments in the other further supports this interrelationship [61]. In particular, lysosomal activity is impaired in the setting of deficient mitochondrial respiration and disruption of endolysosomal trafficking [61]. Along similar lines, depletion or inhibition of apoptosis-inducing factor (AIF), OPA1, or PINK1 in neurons impairs lysosomal activity, thereby inducing accrual of autophagic substrates [62]. Moreover, the restoration of lysosomal pH via the delivery of lysosome-targeted nanoparticles is able to rescue mitophagy in pancreatic β cells exposed to high free fatty acid concentrations [63].

Altogether, these findings indicate that, under certain circumstances, mitochondrial dysfunction develops as a consequence of lysosomal alkalization and that the restoration of lysosomal acidity can reinstate an efficient MQC [63]. In the next paragraph, the possible fate of mildly damaged mitochondria is discussed.

### Generation and Release of Mitochondria-Derived Vesicles

Different from mitophagy, a shuttle system operating via the generation and release of EVs is in place to dispose of mildly damaged mitochondria. This system operates independent of mitochondrial depolarization, autophagy signaling, or mitochondrial fission [64]. Indeed, cells lacking autophagy-related serine/threonine kinase gene (Atg) 5, Beclin-1, or RAB9, as well as silenced for DRP1, are still able to generate MDVs [64].

**Figure 2:**
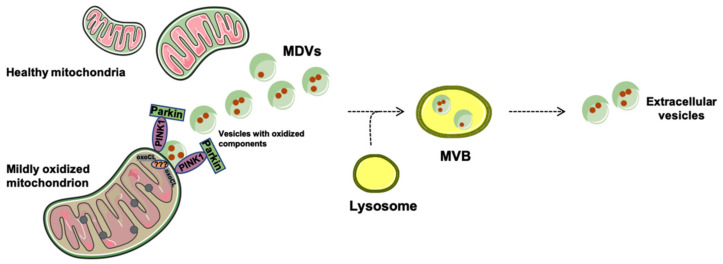
Quality control of mitochondria through the generation and release of mitochondrial-derived vesicles (MDVs). Mildly oxidized mitochondria are also targeted by phosphatase and tensin homolog-induced kinase 1 (PINK1) and Parkin. This priming process, in conjunction with oxidized cardiolipin (oxoCL)-driven membrane curvatures and other unknown proteins, assists in the generation of MDVs. MDVs reach out to the endolysosomal system and form multivesicular bodies (MVBs) that are extruded from cells as extracellular vesicles.

Mitochondrial-enriched small EVs of ~100 nm in diameter [25] are likely delivered to lysosomes for degradation of organellar components [64]. While proceeding in the absence of DRP1, MDV biogenesis seems to require priming by PINK1 and Parkin [25]. Hence, MDV generation may belong to degradative pathways via a system that complements mitophagy for MQC when this degradative process is overwhelmed or compromised [65]. Although the molecular events underlying MDV generation are still unclear, large double-membrane vesicles containing mitochondrial components have been described, providing evidence of crosstalk between mitochondria and the endolysosomal system [6,66]. A proposed mechanism for MDV generation involves the accrual of protein aggregates in proximity to mitochondrial membranes under oxidative stress conditions [25]. This event, concomitant with cardiolipin oxidation, would generate unconventional changes in mitochondrial membrane structure, likely curvatures, that would compete with the function of organelle import channels [25]. The formation of mitochondrial membrane curvatures is thought to be followed by the accumulation of PINK1 at the OMM, followed by ubiquitination and recruitment of Parkin [25]. The process would eventually culminate in the formation of a vesicle which is then released through a process involving unidentified proteins [25] (Figure 2).

Down this road, mitochondrial-lysosomal membrane contact sites finely coordinate the mitochondrial fate between mitophagy and MDV pathways. Indeed, while mitophagy represents an “extreme” attempt of the cell to preserve homeostasis [22,67], MDV generation might dispose of defective mitochondrial components and avoid the clearance of entire organelles [68,69] (Figure 2). Notably, mitochondrial constituents displaced within MDVs (e.g., mtDNA) can activate several inflammatory pathways through the interaction with (1) Toll-like receptors (TLRs), (2) Nod-like receptor (NLR) family pyrin domain containing 3 (NLRP3) inflammasome, and (3) cytosolic cyclic guanosine monophosphate–adenosine monophosphate (GMP–AMP) synthase (cGAS)—stimulator of interferon genes (STING) DNA sensing system [70]. This response is mounted within the framework of innate immunity and pertains to the ‘‘danger theory’’ of inflammation [71]. Indeed, noxious material released from injured cells (i.e., damage-associated molecular patterns (DAMPs)) triggers caspase–1 activation and the secretion of pro-inflammatory cytokines [72]. Of note, PINK and Parkin suppress adaptive immunity responses by favoring the shuttling of oxidized cargo-enriched MDVs to lysosomes for degradation [73]. This prevents the delivery of MDVs to endosomes where mitochondrial components would be loaded on major histocompatibility complex (MHC) class I molecules for antigen presentation [73].

The hypothesis of mitochondrial transfer within MDVs upon functional requests cannot be disregarded. Some lines of evidence indicate that the transfer and uptake of functional mitochondria within MDVs occurs in vitro and in vivo, especially in cells with mitochondrial defects [74,75,76,77]. This event rescues aerobic respiration and suggests a link between mitochondrial defects and endocytosis of EVs [74,75,76,77]. However, whether and how this system contributes to the metabolic regulation of distant cells warrants further investigation.

The persistence of chronic, sterile inflammation is a hallmark of aging [74]. This condition, referred to as “inflamm-aging”, has been shown to contribute to the progression of aging itself and the development of age-related conditions [78,79]. A link among MQC failure, MDV secretion, and inflammation has been hypothesized in several contexts, including progeroid conditions such as human immunodeficiency virus (HIV) infection, a model of accelerated and accentuated aging [80], multiorgan failure [81], inflamm-aging [82], and neurodegeneration [6]. Although the pathophysiology of these conditions is heterogeneous, the release of mitochondrial DAMPs may be a common pathogenic pathway. In this context, the scavenging of circulating mitochondrial DAMPs might represent an unexplored therapeutic option for several disease conditions. Relevant pathways elicited upon MDV release during aging are discussed in the next section.

## 3. Failing Mitochondrial Quality Control and Inflammation during Aging: Partners in Crime

An efficient MQC is pivotal for maintaining cell and organismal homeostasis. Indeed, declines in MQC spanning from mitochondriogenesis to proteostasis via UPR^mt^ have been described during aging and age-associated conditions [23,83]. Alterations in the epigenetic regulation of genes involved in MQC have recently been associated with mitochondrial dyshomeostasis, which may be relevant to aging and chronic degenerative diseases [84]. Furthermore, methylation of specific mtDNA regions may increase susceptibility to neurodegenerative diseases, including AD and PD [85]. Interestingly, mitoepigenetics chromatin remodeling can activate the UPR^mt^ signaling and promote longevity in several eukaryotic species [86,87].

The tight relationship among age-related mitochondrial dysfunction, redox imbalance, and inflammation is indirect evidence of MQC relevance for the cell [88]. Indeed, under specific circumstances, redox-sensitive inflammatory pathways may be activated. This is the case of pathways pertaining to mitochondrial calcium metabolism, iron handling, and reactive oxygen species (ROS) production [89,90]. ROS bursts, in particular, act as major pro-inflammatory stimuli via the activation of nuclear factor κB (NF-κB) [91]. The severity of the inflammatory response and the efficiency of cellular quality control systems determine the cell’s fate. While an apoptotic cascade may be elicited by moderate inflammatory stimuli and overwhelmed cellular repair systems, necrosis may ensue in the setting of overt inflammation, mitochondrial dysfunction, and ROS-induced damage [92]. As a consequence, cell components, including entire and fragmented mitochondria, may be extruded at the systemic level in the form of cell-free molecules or within MDVs. Here, mtDNA and damaged mitochondrial components may act as DAMPs and instigate inflammation by interacting with TLR, NLRP, and cGAS–STING systems [93,94].

DAMPs can engage the TLR pathway that promotes neutrophils recruitment and the instigation of an inflammatory response via NF-κB signaling [95]. Alternatively, mtDNA can induce inflammation via NLRP3 inflammasome activation [96,97]. An NLRP3 inflammatory response has been observed in a variety of conditions, including AD, cardiovascular disease, metabolic disorders, autoimmune diseases, etc. (reviewed in [98]). NLRP3 operates through a set of cytosolic protein complexes that, upon activation, engage caspase-1 and promote caspase-1-dependent cleavage and activation of interleukin (IL) 1 and 18 [99]. The synergistic activity of redox-sensitive inflammatory and inflammasome-mediated pathways concurs to reinforcing inflammation [100].

Albeit the molecular determinants linking inflammasome activation to inflamm-aging remain to be clarified, the presence of bacterial-like motifs within the mtDNA that can be sensed by NLRs are believed to play a major role [101]. Notably, a self-sustaining circle of mitochondrial dysfunction, ROS bursts, and consequent mtDNA damage can be triggered by NLRP3 activators [97]. Among these, oxidized mtDNA, once released, may serve as the ultimate NLRP3 ligand [97]. As such, the activation of inflammasomes, including NLRP3, may be an upstream checkpoint of the innate immune system during the deployment of inflamm-aging. The cGAS–STING DNA-sensing pathway also contributes to sterile inflammation as part of the innate immune system [94]. In particular, the binding of mtDNA to cGAS induces the recruitment of STING protein and the subsequent phosphorylation of the transcription factor, interferon (IFN) regulatory factor 3 (IRF-3) via TRAF family member-associated NF-κB activator (TANK)-binding kinase (TBK). Once activated, IRF-3 triggers the expression of type I and III IFN and IFN-stimulated nuclear genes. When constitutively active, the cGAS–STING pathway promotes inflamm-aging by favoring cellular senescence through IFN-mediated activation of p53 [102,103,104]. Senescent cells, while remaining metabolically active, undergo morphological and functional changes that become manifest with the senescence-associated secretory phenotype (SASP) [105]. This SASP fingerprint encompasses ILs, chemokines, growth factors, secreted proteases, and secreted extracellular matrix components [105]. The release of SASP factors perturbs the local microenvironment through autocrine and paracrine actions aimed at preventing the growth of damaged cells while recruiting immune cells and promoting tissue repair [106,107]. On a different note, insufficient clearance of senescent cells during aging may fuel systemic inflammation via the excessive production of SASP-related pro-inflammatory cytokines (e.g., IL1β, IL6, IL8) [108].

A specific EV SASP (eSASP) has been described [109]. The analysis of exosomes/EV composition showed protein changes in senescent cells that were largely distinct from those of non-senescent matching cells [109]. Moreover, vesicle characterization identified plasma membrane protein signatures of their originating cells [110,111]. Hence, the analysis of eSASP may offer a unique opportunity to identify senescence biomarkers with a degree of cell-type specificity or arising from different stressors.

### Human Immunodeficiency Virus Infection

Inflammation, overreacting innate immunity, and cluster of differentiation (CD)4^+^ T cell depletion are hallmarks of HIV infection [112,113]. HIV infection involves an accelerated aging process and, as such, it confers considerable risk for major geriatric syndromes [80]. A massive release of DAMPs resulting from dysfunctional autophagy and pyroptosis, a highly inflammatory form of programmed cell death, has been proposed as a prominent point of convergence between HIV infection and inflamm-aging [78].

The infectious cycle of HIV is complex and includes an acute and a chronic stage. An early site of HIV exposure and infection is the gut-associated lymphatic tissue (GALT), the largest component of the lymphoid system. The GALT comprises tonsils, Peyer’s patches, lymphoid follicles, and lymphoid cells disseminated throughout the intestinal epithelium and lamina propria [114]. Upon HIV infection, GALT undergoes progressive CD4^+^ T cell depletion, which is due to the apoptosis of infected cells or their elimination by CD8^+^ cytotoxic T cells, as well as to the cell death of bystander uninfected T cells [115,116,117]. At sites of pyroptotic bystander cell death, the release of cellular content triggers inflammation and attracts HIV-susceptible CD4^+^ T cells [112,113]. In turn, inflammation and overreacting type I IFN responses master the chronic phase of the infection. Such changes have also been described in patients on antiretroviral therapy, in whom drug toxicity and an inflamm-aging-like state concur to the pathogenesis of HIV-associated comorbidities [118,119].

Among the mechanisms involved in the complex pathophysiology of HIV, dysfunctional autophagy seems to play a prominent role [120]. This degradative pathway is crucial for cell-autonomous defense against HIV variants void of the negative replication factor (Nef) gene [120]. The viral protein Nef binds to Beclin-1 and blocks the maturation stage of autophagy, thus impeding HIV clearance [121]. A blockade of autophagy has been described during other viral infections. Indeed, the influenza A virus M2 protein and the herpes simplex virus 1 protein ICP34.5 have been shown to target Beclin-1 and to inhibit the late stages of autophagy [122,123].

The finding of Nef-driven inhibition of autophagic maturation might be one of the mechanisms whereby HIV infection triggers inflamm-aging. Indeed, Nef-induced stalling of autophagy is associated with the accrual of multivesicular body endosome (MVB)-like organelles [124,125] and the formation of large vacuoles [126] for all of which a role in the autophagic flux has been hypothesized. In particular, these structures may represent intermediates of the Nef-imposed block to autophagic maturation that could assist in virion assembly and maturation or vehiculate its trafficking instead of supporting its degradation [121]. Even if DNA sensors such as cGAS and various inflammasomes are activated, viral biomolecules represent only a small fraction of the pool of released molecules. Such an observation may find an explanation in the pyroptotic nature of the infection. Accordingly, the massive release of DAMPs, including self-DNA of mitochondrial and genomic origin and DNA-binding proteins such as chaperones and histones, are in the spotlight for triggering HIV-associated inflammation [82].

Studies reported higher levels of mtDNA in plasma of HIV-1-infected individuals [3,127,128] and decreased mitochondrial fitness in regulatory CD4^+^ T cells of immunological non-responders (i.e., patients not showing satisfactory immune recovery under antiretroviral therapy) [129,130]. Circulating genomic DNA (gDNA) and mtDNA acquire immunogenic properties and become accessible to intracellular DNA sensors when bound to chaperones, such as high-mobility group box protein 1, histones, or secreted antimicrobial peptides, as well as when transported into EVs [131]. Hence, mtDNA extruded within EVs or bound to them as part of the DAMPs system has attracted interest as a tool for the detection of mitochondrial dysfunction and associated inflamm-aging [7,79].

Although age-related and HIV-associated chronic inflammation is primed by different mechanisms, the release of mitochondrial DAMPs may be a converging mechanism that could be exploited to develop therapeutics for both conditions.

## 4. Mitochondrial Quality Control in Neurodegeneration

### 4.1. Parkinson’s Disease

PD is a neurodegenerative disease condition with complex pathophysiology that recapitulates all major hallmarks of aging. As such, PD may be envisioned as a prototypical geroscience condition [4,129,132]. In this scenario, mitochondrial dysfunction in neurons and neuroinflammation are considered to be relevant pathogenic mechanisms [133,134]. Although the molecular events linking these two processes are yet elusive, defective MQC and DAMPs release has been suggested as a contributing factor [135]. Notably, the deletion of either *PINK1* or *parkin* gene (*PARK2*) results in a STING-dependent activation of inflammation as a consequence of disruption of autophagic removal of damaged mitochondria [135]. Indeed, Parkin, besides its role as a mitophagy mediator, also functions as a regulator of adaptive immunity by controlling the delivery of mitochondrial antigens to endosomes where they are loaded on MHC class I molecules [73]. Similarly, the mitophagy regulator RAB7A may intervene in the regulation of MDV fusion with late endosome for mitochondrial antigen presentation, as indicated by the accumulation of MDVs in RAB7A knockdown cells [73]. Though, in the absence of PINK1 or Parkin, mitochondrial antigen presentation in immune cells can still occur via the generation and trafficking of MDVs [73]. The loss of PINK1/Parkin in PD would, therefore, result in both MQC disruption and neuroinflammation through MDV-mediated mitochondrial antigen presentation [73].

Recently, mitochondrial DAMPs have been detected within the small EVs purified from the serum of older adults with PD [6]. The EV molecular profile of PD was also characterized by a specific inflammatory signature [6]. In particular, older people with PD had higher serum concentrations of small EVs compared with non-PD controls [6]. The characterization of purified small EVs revealed that they included exosomes of endosomal origin deriving from the fusion of MVBs with the plasma membrane [6]. Furthermore, the identification of mitochondrial signatures within small EVs indicated the presence of circulating MDVs in older adults with PD [6]. Remarkably, lower levels of MDVs were determined in participants with PD relative to non-PD controls [6]. As previously discussed, the generation and release of MDVs are regulated through physical and functional interactions between mitochondria and lysosomes that enable the clearance of damaged mitochondrial constituents [7]. Hence, the increased secretion of small EVs in PD might reflect the cell’s attempt to purge defective mitochondrial components. In turn, the lower secretion of MDVs may indicate a stalling of the MQC system in this condition. Indeed, EV cargoes containing damaged mitochondria may also be re-routed to lysosomes for degradation [25]. Indeed, alterations of lysosomal function were found to be associated with impaired mitochondrial biogenesis in fibroblasts of a young PD patient with *PARK2* mutation [136]. Mutations in the *Vps35* gene have also been reported in late-onset PD individuals [137,138]. Notably, Vps35 protein has been shown to localize at the level of mitochondria and to mediate the generation and release of MDVs targeted to peroxisomes [139]. The reasons for MDVs delivery to peroxisomes is still unclear. Though, *Vps35* mutations induce extensive mitochondrial fragmentation and impairment in mitochondrial function leading to neuronal loss in both in vitro and in vivo systems [138]. Moreover, Parkin has been shown to regulate the retromer complex by modulating the tubular and multivesicular domain organization of late endosomes that ultimately triggers the generation of intraluminal vesicles (ILVs) and exosomes [140]. This is enabled by Parkin-mediated ubiquitination of RAB7A at lysine 38, which affects its binding to the effector RAB-Interacting Lysosomal Protein (RILP) [140]. Along the same line, changes in endosomal morphology and membrane dynamics, as well as and retromer and lysosomal dysfunction, have been found in Parkin-deficient cellular models [141,142,143]. Accelerated ILV formation and increased amounts of CD63-positive intraluminal membranes were also observed in fibroblasts from Parkin-deficient patients, induced pluripotent stem cells (iPS)-derived dopaminergic neurons, and HEK293 cells knockdown for *PARK2* [140].

Finally, the integration of mitochondrial analysis with a multi-marker inflammatory analytical platform revealed a molecular fingerprint in older adults with PD encompassing MDV markers and inflammatory biomolecules [6]. The presence of fibroblast growth factor 21 (FGF21) as part of the molecular signature of PD is particularly relevant. Indeed, FGF21 expression is associated with dysfunctional MQC in neurons and is induced in the brains of murine models of tauopathy and prion disease [144]. Hence, FGF21 may represent a “mitokine” and a biomarker for mitochondrial dysfunction in the brain that warrants investigation [144]. As discussed earlier, these observations highlight the prospect of scavenging circulating mitochondrial DAMPs, including mtDNA, to obtain therapeutic gain in PD.

### 4.2. Alzheimer’s Disease

The accrual of insoluble extracellular amyloid β (Aβ) plaques and intraneuronal Tau tangles, together with neuroinflammation, are pathological hallmarks of AD [145]. The site of initial Aβ aggregation is debated; though, it is proposed that excessive intracellular accumulation may eventually result in neuronal lysis, amyloid leakage, and plaque formation [146]. Indeed, Aβ42, the most pathogenic Aβ peptide, seems to selectively accumulate in the perikaryon of neurons within lysosomes or lysosome-derived structures [146]. Alterations of the endolysosomal system are, therefore, believed to be a major pathogenic pathway in AD. The generation of enlarged and dysfunctional MVBs in the presence of bulky levels of Aβ42 has been described in AD transgenic neurons [147]. In turn, MVB impairment induces Aβ accrual in the enlarged endocytic compartment [147]. Furthermore, enhanced extracellular secretion of amyloid precursor protein (APP) following MVB dysfunction has been found in AD transgenic mice [147]. The proteolytic cleavage of APP protein by β and γ-secretases induces aberrant Aβ production [148]. Amyloidogenesis is also influenced by the intracellular trafficking of APP and the β-site APP-Cleaving-Enzyme (BACE1) and is promoted by the colocalization of APP and BACE1 in the endosomal compartments [149]. Notably, the activity of the retromer complex reduces Aβ production by favoring retrograde transport of APP from endosomes to the TGN [150]. Indeed, impaired retromer complex activity has been involved in AD pathogenesis by promoting higher Aβ production [150].

A role for RAB7A GTPase in AD has also been shown. A higher expression of RAB7A has been found in the frontal cortex of transgenic mice with a progressive form of AD [151], while RAB7A depletion is able to reduce Tau secretion [152]. Hence, RAB7A may contribute to the extracellular accumulation of pathological Tau species and support the propagation of Tau pathology in AD [152]. This hypothesis is in line with the observations of decreased or increased Tau secretion following the expression of a dominant-negative (RAB7A T22N) or a constitutively active (RAB7 Q67L) RAB7A mutant, respectively [152]. The dual role of RAB7A in the regulation of mitophagy and Tau secretion might, therefore, suggest that the intracellular trafficking mediated by RAB7A may represent a relevant factor in the pathogenesis of AD.

Defective MQC processes, in particular mitophagy, are considered to be the trait d’union linking mitochondrial dysfunction and bioenergetic failure in neurons, inflammation, and eventually neuronal loss [153]. Mitochondrial dysfunction and defective mitophagy have been described in post-mortem hippocampus neurons of AD patients, in induced pluripotent stem cell-derived human AD neurons, and in animal models of AD [154]. Strikingly, the restoration of neuronal mitophagy has been shown to promote the elimination of defective mitochondria, improve mitochondrial bioenergetics, and ameliorate cognitive decline in *Caenorhabditis elegans* models of AD and in the amyloid precursor protein/presenilin 1 APP/PS1 transgenic mouse AD model [154].

A vicious circle between generation Aβ plaque generation and disruption of neuronal mitophagy has been hypothesized [153,154,155]. This pathway might operate through impairment of the mitochondrial unfolded protein response (UPR^mt^) machinery, a component of the mitochondrial proteostasis system, that is believed to link mitochondrial dyshomeostasis to Aβ proteotoxicity [156]. The trafficking of the activating transcription factor associated with stress (ATFS-1) may play a relevant role in the process [157]. Under physiologic conditions, ATFS-1 is imported within the mitochondrial matrix and inhibits the UPR^mt^ [157]. In the setting of mitochondrial dysfunction, instead, ATFS-1 is relocated into the nucleus where it triggers the expression of genes involved in mitochondrial turnover [157]. A lower expression of DISC1 has also been detected in postmortem brain regions of AD patients, transgenic AD mice model, and Aβ-treated cultured cells [158]. Noticeably, DISC1 functions as a mitophagy receptor containing an LC3-interacting region that promotes mitophagy through its binding to LC3 and protects synaptic plasticity from Aβ accumulation-induced toxicity, [158]. The phosphorylated-Tau (p-Tau) protein is another player in the vicious circle between defective mitophagy and mitochondrial dysfunction [153]. The expression of human wild-type (hTau) and frontotemporal dementia mutant Tau (hP301L) inhibits Parkin-dependent mitophagy by reducing or blocking the mitochondrial translocation of Parkin in neuroblastoma cells and *Caenorhabditis elegans*, respectively [159].

The relevance of defective mitophagy to the pathogenesis of AD is further supported by the amelioration of cognitive dysfunction and Aβ proteinopathy in APP/PS1 mice following the restoration of neuronal and microglial mitophagy via supplementation with nicotinamide adenine dinucleotide (NAD^+^) precursors, urolithin A, and actinonin [160]. The pharmacological restoration of mitophagy has also been shown to reduce phosphorylation of Tau at several serine and threonine residues and to mitigate cytokine-induced inflammation ignited by immune activation of microglia [154].

Signs of neuroinflammation (e.g., inflammatory cytokines in the proximity of Aβ deposits and neurofibrillary tangles) have been found in AD [161]. In particular, IL1β, IL6, and tumor necrosis factor alpha (TNF-α) are produced in response to amyloid plaque deposition and are involved in local inflammation [162]. Sustained production of these cytokines causes neurotoxicity and promotes the generation of Aβ peptides [162]. Impairments in mitophagy and the ensuing mitochondrial dysfunction observed in AD may underpin the generation and release of DAMPs with consequent stimulation of innate immunity by TLR, NLRP, and cGAS–STING systems [93,94]. Similar to PD [6], mitochondrial DAMPs might be released within MDVs also in AD. Should this pathway be operative, it may provide a mechanistic explanation for the state of systemic inflammation observed in AD [163]. Furthermore, in-depth characterization of the cargo of circulating MDVs might reveal novel biomarkers to be used for the identification and monitoring of AD patients.

## 5. Mitochondrial Quality Control in Cardiovascular Disease

Dysmorphic mitochondria producing high levels of ROS have been found in aged cardiomyocytes [164] in association with cardiac structural and functional alterations [165]. These observations laid the ground for the study of mitochondrial dysfunction and inefficient MQC as mechanisms in heart senescence [5].

The heart is one of the most robust mitophagy organs relying upon this degradative process for normal functioning [166]. Cardiac autophagy, either selective or unselective, is modulated by cardiomyocyte energy status via metabolic signaling. Under substrate deficit or oxidative stress conditions, the drop of intramyocyte ATP activates an energy-sensing pathway involving 5′-AMP-activated protein kinase (AMPK), unc-51-like kinase 1 (ULK1), BCL2, and Beclin-1 [167,168]. The downregulation of the mechanistic target of rapamycin complex 1 (mTORC1), an autophagy suppressor, further ignites autophagy [169]. To avoid excessive degradation, growth, or cell survival, stimuli convey anti-autophagic signaling through insulin/insulin-like growth factor 1 (IGF1)/protein kinase B (AKT1) signaling [170]. In addition, the GTP-binding protein Ras homolog enriched in brain (RHEB) represses autophagy primarily via mTORC1 and transcription factors related to lysosomal biogenesis (i.e., transcription factor EB (TFEB)) [171].

With regard to mitophagy, both PINK-Parkin-dependent and independent pathways have been described in cardiomyocytes [172]. While canonical PINK-Parkin-dependent mitophagy preserves mitochondrial and cardiac function in diabetic mice with high-fat diet-induced cardiomyopathy [173], defects in PINK-Parkin-dependent mitophagy leads to severe cardiac complications in animal models of Duchenne muscular dystrophy [172]. The expression of negative regulators of cardiomyocyte mitophagy, such as the mammalian Ste20-like kinase 1 (Mst1), has also been documented in an experimental model of septic cardiomyopathy [174]. Notably, Mst1 depletion attenuates lipopolysaccharide (LPS)-induced cardiomyocyte death and preserves cardiac function via promoting Parkin-dependent mitophagy [174]. Moreover, a role for the adenine nucleotide translocator (ANT) complex in mitophagy regulation via interaction with TIM23 has been described [175]. This ANT function is independent of the TIM23-mediated protein translocator and results in the stabilization of PINK1 at damaged mitochondria for subsequent degradation [175]. The depletion of ANT in the murine heart causes accrual of dysmorphic mitochondria, cardiomyocyte hypertrophy, and contractile dysfunction [175]. Notably, patients with homozygous mutations in ANT1 show severe heart failure in conjunction with cardiac mitochondrial dyshomeostasis [175].

PINK1-Parkin-independent mitophagy, possibly operating via inter-organelle contact sites, has also been reported in the context of CVD. In particular, the mitochondrial receptor FUNDC1 mediates the release of Ca^2+^ from the endoplasmic reticulum (ER) by establishing mitochondrial−ER contacts through the binding of the ER-inositol 1,4,5-trisphosphate type 2 receptor (IP3R2) [176]. These inter-organelle contacts are necessary for maintaining fully-functioning mitochondria and preserving cardiac function [176]. Notably, FUNDC1 expression and FUNDC1-induced mitophagy are repressed by the negative mitophagy regulator Mst1 [177]. In particular, while Mst1 expression is associated with cardiac ischemia-reperfusion injury, its depletion preserves mitochondrial and cardiomyocyte homeostasis [177]. In addition, BNIP3 can trigger mitophagy in cardiomyocytes independent of cytosolic Ca^2+^ levels, oxidative stress, and apoptosis signaling [178]. This action is mediated by phosphorylation of BNIP3 at Ser17 and Ser24, which favors its interaction with LC3, thereby fueling the mitophagy flux [179]. Of note, the downregulation of BNIP3 via abrogation of c-Jun N-terminal kinase (JNK) signaling has been found to reverse cardiac remodeling in heart failure [180].

The induction of UPR^mt^ has been shown to mitigate mitochondrial and contractile dysfunction in cardiomyocytes during pressure overload [181]. Remarkably, the severity of heart failure, as well as the extent of cardiomyocyte apoptosis and cardiac fibrosis, were found to be attenuated in patients with aortic valve stenosis with higher RNA expression of UPR^mt^ mediators [181]. A cardioprotective role has also been hypothesized for RAB7A [182]. Indeed, the stimulation of the mitochondrial aldehyde dehydrogenase 2 (ALDH2) activity has been shown to upregulate autophagy via RAB7A, thereby mitigating oxidative damage in the aged heart [183].

Finally, MDV generation has been proposed as a mechanism to dispose of mildly oxidized mitochondria independent of mitochondrial depolarization, autophagy signaling, or mitochondrial fission [184]. In cultured H9c2 myoblasts, MDV formation acts as a basal housekeeping process and occurs more frequently than mitophagy events [184]. MDV generation is upregulated following exposure to mitochondrial stressors (i.e., antimycin-A and xanthine/xanthine oxidase), while both MDV production and mitophagy are enhanced in response to extensive mitochondrial damage [184]. Although preliminary, these findings suggest that constitutive MDV generation is necessary for the maintenance of cardiomyocyte homeostasis under physiologic conditions and also serves as a first-line defense against mild stressors.

## References

[1] Friedman J.R., Nunnari J. (2014). Mitochondrial form and function. Nature.

[2] Zhang H., Menzies K.J., Auwerx J. (2018). The role of mitochondria in stem cell fate and aging. Development.

[3] Cossarizza A., Pinti M., Nasi M., Gibellini L., Manzini S., Roat E., Biasi S., De Bertoncelli L., Montagna J.P., Bisi L. (2011). Increased plasma levels of extracellular mitochondrial DNA during HIV infection: A new role for mitochondrial damage-associated molecular patterns during inflammation. Mitochondrion.

[4] Guerra F., Arbini A.A., Moro L. (2017). Mitochondria and cancer chemoresistance. Biochim. Biophys. Acta Bioenerg..

[5] Picca A., Mankowski R.T., Burman J.L., Donisi L., Kim J.-S., Marzetti E., Leeuwenburgh C. (2018). Mitochondrial quality control mechanisms as molecular targets in cardiac ageing. Nat. Rev. Cardiol..

[6] Picca A., Guerra F., Calvani R., Marini F., Biancolillo A., Landi G., Beli R., Landi F., Bernabei R., Bentivoglio A. (2020). Mitochondrial signatures in circulating extracellular vesicles of older adults with Parkinson’s disease: Results from the EXosomes in PArkiNson’s Disease (EXPAND) Study. J. Clin. Med..

[7] Picca A., Guerra F., Calvani R., Bucci C., Lo Monaco M.R., Bentivoglio A.R., Coelho-Júnior H.J., Landi F., Bernabei R., Marzetti E. (2019). Mitochondrial dysfunction and aging: Insights from the analysis of extracellular vesicles. Int. J. Mol. Sci..

[8] Pradella L.M., Zuntini R., Magini P., Ceccarelli C., Neri I., Cerasoli S., Graziano C., Gasparre G., Turchetti D. (2011). Two distinct thyroid tumours in a patient with Cowden syndrome carrying both a 10q23 and a mitochondrial DNA germline deletion. J. Med. Genet..

[9] Daniele T., Schiaffino M.V. (2014). Organelle biogenesis and interorganellar connections: Better in contact than in isolation. Commun. Integr. Biol..

[10] Todkar K., Ilamathi H.S., Germain M. (2017). Mitochondria and lysosomes: Discovering bonds. Front. Cell Dev. Biol..

[11] Picca A., Calvani R., Coelho-Junior H.J., Landi F., Bernabei R., Marzetti E. (2020). Inter-organelle membrane contact sites and mitochondrial quality control during aging: A geroscience view. Cells.

[12] Das A., Nag S., Mason A.B., Barroso M.M. (2016). Endosome-mitochondria interactions are modulated by iron release from transferrin. J. Cell Biol..

[13] Hamdi A., Roshan T.M., Kahawita T.M., Mason A.B., Sheftel A.D., Ponka P. (2016). Erythroid cell mitochondria receive endosomal iron by a “kiss-and-run” mechanism. Biochim. Biophys. Acta Mol. Cell Res..

[14] Aston D., Capel R.A., Ford K.L., Christian H.C., Mirams G.R., Rog-Zielinska E.A., Kohl P., Galione A., Burton R.A.B., Terrar D.A. (2017). High resolution structural evidence suggests the sarcoplasmic reticulum forms microdomains with scidic Stores (lysosomes) in the heart. Sci. Rep..

[15] Wong Y.C., Ysselstein D., Krainc D. (2018). Mitochondria-lysosome contacts regulate mitochondrial fission via RAB7 GTP hydrolysis. Nature.

[16] Soto-Heredero G., Baixauli F., Mittelbrunn M. (2017). Interorganelle communication between mitochondria and the endolysosomal system. Front. Cell Dev. Biol..

[17] Jovaisaite V., Mouchiroud L., Auwerx J. (2014). The mitochondrial unfolded protein response, a conserved stress response pathway with implications in health and disease. J. Exp. Biol..

[18] Stach L., Freemont P.S. (2017). The AAA+ ATPase p97, a cellular multitool. Biochem. J..

[19] Zhao Q., Wang J., Levichkin I.V., Stasinopoulos S., Ryan M.T., Hoogenraad N.J. (2002). A mitochondrial specific stress response in mammalian cells. EMBO J..

[20] Fiorese C.J., Schulz A.M., Lin Y.F., Rosin N., Pellegrino M.W., Haynes C.M. (2016). The transcription factor ATF5 mediates a mammalian mitochondrial UPR. Curr. Biol..

[21] Twig G., Hyde B., Shirihai O.S. (2008). Mitochondrial fusion, fission and autophagy as a quality control axis: The bioenergetic view. Biochim. Biophys. Acta.

[22] Youle R.J., Narendra D.P. (2011). Mechanisms of mitophagy. Nat. Rev. Mol. Cell Biol..

[23] Picca A., Lezza A.M.S. (2015). Regulation of mitochondrial biogenesis through TFAM-mitochondrial DNA interactions. Useful insights from aging and calorie restriction studies. Mitochondrion.

[24] Miyamoto Y., Kitamura N., Nakamura Y., Futamura M., Miyamoto T., Yoshida M., Ono M., Ichinose S., Arakawa H. (2011). Possible existence of lysosome-like organella within mitochondria and its role in mitochondrial quality control. PLoS ONE.

[25] Sugiura A., McLelland G.-L., Fon E.A., McBride H.M. (2014). A new pathway for mitochondrial quality control: Mitochondrial-derived vesicles. EMBO J..

[26] Terman A., Kurz T., Navratil M., Arriaga E.A., Brunk U.T. (2010). Mitochondrial turnover and aging of long-lived postmitotic cells: The mitochondrial-lysosomal axis theory of aging. Antioxid. Redox Signal..

[27] Mishra P., Chan D.C. (2016). Metabolic regulation of mitochondrial dynamics. J. Cell Biol..

[28] Lewis S.C., Uchiyama L.F., Nunnari J. (2016). ER-mitochondria contacts couple mtDNA synthesis with mitochondrial division in human cells. Science.

[29] Smirnova E., Griparic L., Shurland D.L., Van der Bliek A.M. (2001). Dynamin-related protein Drp1 is required for mitochondrial division in mammalian cells. Mol. Biol. Cell.

[30] Friedman J.R., Lackner L.L., West M., DiBenedetto J.R., Nunnari J., Voeltz G.K. (2011). ER tubules mark sites of mitochondrial division. Science.

[31] Moore A.S., Wong Y.C., Simpson C.L., Holzbaur E.L.F. (2016). Dynamic actin cycling through mitochondrial subpopulations locally regulates the fission-fusion balance within mitochondrial networks. Nat. Commun..

[32] Lee J.E., Westrate L.M., Wu H., Page C., Voeltz G.K. (2016). Multiple dynamin family members collaborate to drive mitochondrial division. Nature.

[33] Yao R.Q., Ren C., Xia Z.F., Yao Y.M. (2020). Organelle-specific autophagy in inflammatory diseases: A potential therapeutic target underlying the quality control of multiple organelles. Autophagy.

[34] Narendra D., Tanaka A., Suen D.F., Youle R.J. (2008). Parkin is recruited selectively to impaired mitochondria and promotes their autophagy. J. Cell Biol..

[35] Kazlauskaite A., Muqit M.M.K. (2015). PINK1 and Parkin—mitochondrial interplay between phosphorylation and ubiquitylation in Parkinson’s disease. FEBS J..

[36] Aerts L., Craessaerts K., De Strooper B., Morais V.A. (2015). PINK1 kinase catalytic activity is regulated by phosphorylation on serines 228 and 402. J. Biol. Chem..

[37] Koyano F., Matsuda N. (2015). Molecular mechanisms underlying PINK1 and Parkin catalyzed ubiquitylation of substrates on damaged mitochondria. Biochim. Biophys. Acta.

[38] Narendra D.P., Jin S.M., Tanaka A., Suen D.F., Gautier C.A., Shen J., Cookson M.R., Youle R.J. (2010). PINK1 is selectively stabilized on impaired mitochondria to activate Parkin. PLoS Biol..

[39] Matsuda N., Sato S., Shiba K., Okatsu K., Saisho K., Gautier C.A., Sou Y.-S., Saiki S., Kawajiri S., Sato F. (2010). PINK1 stabilized by mitochondrial depolarization recruits Parkin to damaged mitochondria and activates latent Parkin for mitophagy. J. Cell Biol..

[40] Vives-Bauza C., Zhou C., Huang Y., Cui M., de Vries R.L.A., Kim J., May J., Tocilescu M.A., Liu W., Ko H.S. (2010). PINK1-dependent recruitment of Parkin to mitochondria in mitophagy. Proc. Natl. Acad. Sci. USA.

[41] Kondapalli C., Kazlauskaite A., Zhang N., Woodroof H.I., Campbell D.G., Gourlay R., Burchell L., Walden H., Macartney T.J., Deak M. (2012). PINK1 is activated by mitochondrial membrane potential depolarization and stimulates Parkin E3 ligase activity by phosphorylating Serine 65. Open Biol..

[42] Shiba-Fukushima K., Imai Y., Yoshida S., Ishihama Y., Kanao T., Sato S., Hattori N. (2012). PINK1-mediated phosphorylation of the Parkin ubiquitin-like domain primes mitochondrial translocation of Parkin and regulates mitophagy. Sci. Rep..

[43] Wong Y.C., Holzbaur E.L.F. (2014). Optineurin is an autophagy receptor for damaged mitochondria in parkin-mediated mitophagy that is disrupted by an ALS-linked mutation. Proc. Natl. Acad. Sci. USA.

[44] Lazarou M., Sliter D.A., Kane L.A., Sarraf S.A., Wang C., Burman J.L., Sideris D.P., Fogel A.I., Youle R.J. (2015). The ubiquitin kinase PINK1 recruits autophagy receptors to induce mitophagy. Nature.

[45] Geisler S., Holmström K.M., Skujat D., Fiesel F.C., Rothfuss O.C., Kahle P.J., Springer W. (2010). PINK1/Parkin-mediated mitophagy is dependent on VDAC1 and p62/SQSTM1. Nat. Cell Biol..

[46] Bowling J.L., Skolfield M.C., Riley W.A., Nolin A.P., Wolf L.C., Nelson D.E. (2019). Temporal integration of mitochondrial stress signals by the PINK1:Parkin pathway. BMC Mol. Cell Biol..

[47] Kolitsida P., Zhou J., Rackiewicz M., Nolic V., Dengjel J., Abeliovich H. (2019). Phosphorylation of mitochondrial matrix proteins regulates their selective mitophagic degradation. Proc. Natl. Acad. Sci. USA.

[48] Tan E., Tang B. (2019). Rab7a and mitophagosome formation. Cells.

[49] Guerra F., Bucci C. (2016). Multiple roles of the small GTPase Rab7. Cells.

[50] Guerra F., Bucci C. (2019). Role of the RAB7 protein in tumor progression and cisplatin chemoresistance. Cancers (Basel).

[51] Yamano K., Fogel A.I., Wang C., van der Bliek A.M., Youle R.J. (2014). Mitochondrial Rab GAPs govern autophagosome biogenesis during mitophagy. Elife.

[52] Pankiv S., Alemu E.A., Brech A., Bruun J.-A., Lamark T., Overvatn A., Bjørkøy G., Johansen T. (2010). FYCO1 is a Rab7 effector that binds to LC3 and PI3P to mediate microtubule plus end-directed vesicle transport. J. Cell Biol..

[53] Jäger S., Bucci C., Tanida I., Ueno T., Kominami E., Saftig P., Eskelinen E.-L. (2004). Role for Rab7 in maturation of late autophagic vacuoles. J. Cell Sci..

[54] Gutierrez M.G., Munafó D.B., Berón W., Colombo M.I. (2004). Rab7 is required for the normal progression of the autophagic pathway in mammalian cells. J. Cell Sci..

[55] Zhao T., Huang X., Han L., Wang X., Cheng H., Zhao Y., Chen Q., Chen J., Cheng H., Xiao R. (2012). Central role of mitofusin 2 in autophagosome-lysosome fusion in cardiomyocytes. J. Biol. Chem..

[56] Jimenez-Orgaz A., Kvainickas A., Nägele H., Denner J., Eimer S., Dengjel J., Steinberg F. (2018). Control of RAB 7 activity and localization through the retromer-TBC1D5 complex enables RAB7-dependent mitophagy. EMBO J..

[57] Burd C., Cullen P.J. (2014). Retromer: A master conductor of endosome sorting. Cold Spring Harb. Perspect. Biol..

[58] Jia D., Zhang J.S., Li F., Wang J., Deng Z., White M.A., Osborne D.G., Phillips-Krawczak C., Gomez T.S., Li H. (2016). Structural and mechanistic insights into regulation of the retromer coat by TBC1d5. Nat. Commun..

[59] Lin X., Zhang J., Chen L., Chen Y., Xu X., Hong W., Wang T. (2017). Tyrosine phosphorylation of Rab7 by Src kinase. Cell. Signal..

[60] Heo J.M., Ordureau A., Swarup S., Paulo J.A., Shen K., Sabatini D.M., Harper J.W. (2018). RAB7A phosphorylation by TBK1 promotes mitophagy via the PINK-PARKIN pathway. Sci. Adv..

[61] Baixauli F., Acín-Pérez R., Villarroya-Beltrí C., Mazzeo C., Nuñez-Andrade N., Gabandé-Rodriguez E., Ledesma M.D., Blázquez A., Martin M.A., Falcón-Pérez J.M. (2015). Mitochondrial respiration controls lysosomal function during inflammatory T cell responses. Cell Metab..

[62] Demers-Lamarche J., Guillebaud G., Tlili M., Todkar K., Bélanger N., Grondin M., Nguyen A.P., Michel J., Germain M. (2016). Loss of mitochondrial function impairs lysosomes. J. Biol. Chem..

[63] Assali E.A., Shlomo D., Zeng J., Taddeo E.P., Trudeau K.M., Erion K.A., Colby A.H., Grinstaff M.W., Liesa M., Las G. (2018). Nanoparticle-mediated lysosomal reacidification restores mitochondrial turnover and function in β cells under lipotoxicity. FASEB J..

[64] Soubannier V., McLelland G.-L., Zunino R., Braschi E., Rippstein P., Fon E.A., McBride H.M. (2012). A vesicular transport pathway shuttles cargo from mitochondria to lysosomes. Curr. Biol..

[65] Desdín-Micó G., Mittelbrunn M. (2017). Role of exosomes in the protection of cellular homeostasis. Cell Adhes. Migr..

[66] Sansone P., Savini C., Kurelac I., Chang Q., Amato L.B., Strillacci A., Stepanova A., Iommarini L., Mastroleo C., Daly L. (2017). Packaging and transfer of mitochondrial DNA via exosomes regulate escape from dormancy in hormonal therapy-resistant breast cancer. Proc. Natl. Acad. Sci. USA.

[67] Roberts R.F., Tang M.Y., Fon E.A., Durcan T.M. (2016). Defending the mitochondria: The pathways of mitophagy and mitochondrial-derived vesicles. Int. J. Biochem. Cell Biol..

[68] McLelland G.-L., Soubannier V., Chen C.X., McBride H.M., Fon E.A. (2014). Parkin and PINK1 function in a vesicular trafficking pathway regulating mitochondrial quality control. EMBO J..

[69] Soubannier V., Rippstein P., Kaufman B.A., Shoubridge E.A., McBride H.M. (2012). Reconstitution of mitochondria derived vesicle formation demonstrates selective enrichment of oxidized cargo. PLoS ONE.

[70] Picca A., Lezza A.M.S., Leeuwenburgh C., Pesce V., Calvani R., Bossola M., Manes-Gravina E., Landi F., Bernabei R., Marzetti E. (2018). Circulating mitochondrial DNA at the crossroads of mitochondrial dysfunction and inflammation during aging and muscle wasting disorders. Rejuvenation Res..

[71] Zhang Q., Raoof M., Chen Y., Sumi Y., Sursal T., Junger W., Brohi K., Itagaki K., Hauser C.J. (2010). Circulating mitochondrial DAMPs cause inflammatory responses to injury. Nature.

[72] Krysko D.V., Agostinis P., Krysko O., Garg A.D., Bachert C., Lambrecht B.N., Vandenabeele P. (2011). Emerging role of damage-associated molecular patterns derived from mitochondria in inflammation. Trends Immunol..

[73] Matheoud D., Sugiura A., Bellemare-Pelletier A., Laplante A., Rondeau C., Chemali M., Fazel A., Bergeron J.J., Trudeau L.E., Burelle Y. (2016). Parkinson’s disease-related proteins PINK1 and Parkin repress mitochondrial antigen presentation. Cell.

[74] Islam M.N., Das S.R., Emin M.T., Wei M., Sun L., Westphalen K., Rowlands D.J., Quadri S.K., Bhattacharya S., Bhattacharya J. (2012). Mitochondrial transfer from bone-marrow-derived stromal cells to pulmonary alveoli protects against acute lung injury. Nat. Med..

[75] Dong L.F., Kovarova J., Bajzikova M., Bezawork-Geleta A., Svec D., Endaya B., Sachaphibulkij K., Coelho A.R., Sebkova N., Ruzickova A. (2017). Horizontal transfer of whole mitochondria restores tumorigenic potential in mitochondrial DNA-deficient cancer cells. Elife.

[76] Spees J.L., Olson S.D., Whitney M.J., Prockop D.J. (2006). Mitochondrial transfer between cells can rescue aerobic respiration. Proc. Natl. Acad. Sci. USA.

[77] Griessinger E., Moschoi R., Biondani G., Peyron J.-F. (2017). Mitochondrial transfer in the leukemia microenvironment. Trends Cancer.

[78] Franceschi C., Garagnani P., Parini P., Giuliani C., Santoro A. (2018). Inflammaging: A new immune–metabolic viewpoint for age-related diseases. Nat. Rev. Endocrinol..

[79] Picca A., Lezza A.M.S., Leeuwenburgh C., Pesce V., Calvani R., Landi F., Bernabei R., Marzetti E. (2017). Fueling inflamm-aging through mitochondrial dysfunction: Mechanisms and molecular targets. Int. J. Mol. Sci..

[80] Cesari M., Marzetti E., Canevelli M., Guaraldi G. (2017). Geriatric syndromes: How to treat. Virulence.

[81] Aswani A., Manson J., Itagaki K., Chiazza F., Collino M., Wupeng W.L., Chan T.K., Wong W.S.F., Hauser C.J., Thiemermann C. (2018). Scavenging circulating mitochondrial DNA as a potential therapeutic option for multiple organ dysfunction in trauma hemorrhage. Front. Immunol..

[82] Heil M., Brockmeyer N.H. (2019). Self-DNA sensing fuels HIV-1-associated inflammation. Trends Mol. Med..

[83] Shpilka T., Haynes C.M. (2018). The mitochondrial UPR: Mechanisms, physiological functions and implications in ageing. Nat. Rev. Mol. Cell Biol..

[84] D’Aquila P., Montesanto A., Rango F.D., Guarasci F., Passarino G., Bellizzi D. (2019). Epigenetic signature: Implications for mitochondrial quality control in human aging. Aging.

[85] Blanch M., Mosquera J.L., Ansoleaga B., Ferrer I., Barrachina M. (2016). Altered mitochondrial DNA methylation pattern in Alzheimer disease-related pathology and in Parkinson disease. Am. J. Pathol..

[86] Merkwirth C., Jovaisaite V., Durieux J., Matilainen O., Jordan S.D., Quiros P.M., Steffen K.K., Williams E.G., Mouchiroud L., Tronnes S.U. (2016). Two conserved histone demethylases regulate mitochondrial stress-induced longevity. Cell.

[87] Tian Y., Garcia G., Bian Q., Steffen K.K., Joe L., Wolff S., Meyer B.J., Dillin A. (2016). mitochondrial stress induces chromatin reorganization to promote longevity and UPRmt. Cell.

[88] López-Armada M.J., Riveiro-Naveira R.R., Vaamonde-García C., Valcárcel-Ares M.N. (2013). Mitochondrial dysfunction and the inflammatory response. Mitochondrion.

[89] Maass D.L., White J., Sanders B., Horton J.W. (2005). Role of cytosolic vs. mitochondrial Ca^2+^ accumulation in burn injury-related myocardial inflammation and function. Am. J. Physiol. Heart Circ. Physiol..

[90] Picca A., Mankowski R.T., Kamenov G., Anton S.D., Manini T.M., Buford T.W., Saini S.K., Calvani R., Landi F., Bernabei R. (2019). Advanced age is associated with iron dyshomeostasis and mitochondrial DNA damage in human skeletal muscle. Cells.

[91] Schreck R., Rieber P., Baeuerle P.A. (1991). Reactive oxygen intermediates as apparently widely used messengers in the activation of the NF-kappa B transcription factor and HIV-1. EMBO J..

[92] Fiers W., Beyaert R., Declercq W., Vandenabeele P. (1999). More than one way to die: Apoptosis, necrosis and reactive oxygen damage. Oncogene.

[93] Collins L.V., Hajizadeh S., Holme E., Jonsson I.-M., Tarkowski A. (2004). Endogenously oxidized mitochondrial DNA induces in vivo and in vitro inflammatory responses. J. Leukoc. Biol..

[94] Cai X., Chiu Y.H., Chen Z.J. (2014). The cGAS-cGAMP-STING pathway of cytosolic DNA sensing and signaling. Mol. Cell.

[95] Takeuchi O., Akira S. (2010). Pattern recognition receptors and inflammation. Cell.

[96] Zhou R., Yazdi A.S., Menu P., Tschopp J. (2011). A role for mitochondria in NLRP3 inflammasome activation. Nature.

[97] Shimada K., Crother T.R., Karlin J., Dagvadorj J., Chiba N., Chen S., Ramanujan V.K., Wolf A.J., Vergnes L., Ojcius D.M. (2012). Oxidized mitochondrial DNA activates the NLRP3 inflammasome during apoptosis. Immunity.

[98] Mangan M.S.J., Olhava E.J., Roush W.R., Seidel H.M., Glick G.D., Latz E. (2018). Targeting the NLRP3 inflammasome in inflammatory diseases. Nat. Rev. Drug Discov..

[99] Martinon F., Burns K., Tschopp J. (2002). The inflammasome: A molecular platform triggering activation of inflammatory caspases and processing of proIL-β. Mol. Cell.

[100] Strowig T., Henao-Mejia J., Elinav E., Flavell R. (2012). Inflammasomes in health and disease. Nature.

[101] Zhong Z., Liang S., Sanchez-Lopez E., He F., Shalapour S., Lin X., Wong J., Ding S., Seki E., Schnabl B. (2018). New mitochondrial DNA synthesis enables NLRP3 inflammasome activation. Nature.

[102] Moiseeva O., Mallette F.A., Mukhopadhyay U.K., Moores A., Ferbeyre G. (2006). DNA damage signaling and p53-dependent senescence after prolonged β-interferon stimulation. Mol. Biol. Cell.

[103] Glück S., Guey B., Gulen M.F., Wolter K., Kang T.-W., Schmacke N.A., Bridgeman A., Rehwinkel J., Zender L., Ablasser A. (2017). Innate immune sensing of cytosolic chromatin fragments through cGAS promotes senescence. Nat. Cell Biol..

[104] Yang H., Wang H., Ren U., Chen Q., Chena Z.J. (2017). CGAS is essential for cellular senescence. Proc. Natl. Acad. Sci. USA.

[105] Coppé J.-P., Patil C.K., Rodier F., Sun Y., Muñoz D.P., Goldstein J., Nelson P.S., Desprez P.-Y., Campisi J. (2008). Senescence-associated secretory phenotypes reveal cell-nonautonomous functions of oncogenic RAS and the p53 tumor suppressor. PLoS Biol..

[106] Acosta J.C., Banito A., Wuestefeld T., Georgilis A., Janich P., Morton J.P., Athineos D., Kang T.-W., Lasitschka F., Andrulis M. (2013). A complex secretory program orchestrated by the inflammasome controls paracrine senescence. Nat. Cell Biol..

[107] Scheibye-Knudsen M., Fang E.F., Croteau D.L., Wilson D.M., Bohr V.A. (2015). Protecting the mitochondrial powerhouse. Trends Cell Biol..

[108] Watanabe S., Kawamoto S., Ohtani N., Hara E. (2017). The impact of SASP and its potential as a therapeutic target for senescence-associated diseases. Cancer Sci..

[109] Basisty N., Kale A., Jeon O.H., Kuehnemann C., Payne T., Rao C., Holtz A., Shah S., Sharma V., Ferrucci L. (2020). A proteomic atlas of senescence-associated secretomes for aging biomarker development. PLoS Biol..

[110] Takasugi M. (2018). Emerging roles of extracellular vesicles in cellular senescence and aging. Aging Cell.

[111] Belov L., Matic K.J., Hallal S., Best O.G., Mulligan S.P., Christopherson R.I. (2016). Extensive surface protein profiles of extracellular vesicles from cancer cells may provide diagnostic signatures from blood samples. J. Extracell. Vesicles.

[112] Doitsh G., Greene W.C. (2016). Dissecting how CD4 T cells are lost during HIV Infection. Cell Host Microbe.

[113] Doitsh G., Galloway N.L.K., Geng X., Yang Z., Monroe K.M., Zepeda O., Hunt P.W., Hatano H., Sowinski S., Muñoz-Arias I. (2014). Cell death by pyroptosis drives CD4 T-cell depletion in HIV-1 infection. Nature.

[114] Feria M.G., Taborda N.A., Hernandez J.C., Rugeles M.T. (2018). HIV replication is associated to inflammasomes activation, IL-1β, IL-18 and caspase-1 expression in GALT and peripheral blood. PLoS ONE.

[115] Perfettini J.L., Castedo M., Roumier T., Andreau K., Nardacci R., Piacentini M., Kroemer G. (2005). Mechanisms of apoptosis induction by the HIV-1 envelope. Cell Death Differ..

[116] Muthumani K., Choo A.Y., Hwang D.S., Premkumar A., Dayes N.S., Harris C., Green D.R., Wadsworth S.A., Siekierka J.J., Weiner D.B. (2005). HIV-1 Nef-induced FasL induction and bystander killing requires p38 MAPK activation. Blood.

[117] Varbanov M., Espert L., Biard-Piechaczyk M. (2006). Mechanisms of CD4 T-cell depletion triggered by HIV-1 viral proteins. AIDS Rev..

[118] Nasi M., De Biasi S., Gibellini L., Bianchini E., Pecorini S., Bacca V., Guaraldi G., Mussini C., Pinti M., Cossarizza A. (2017). Ageing and inflammation in patients with HIV infection. Clin. Exp. Immunol..

[119] Nasi M., Pinti M., Mussini C., Cossarizza A. (2014). Persistent inflammation in HIV infection: Established concepts, new perspectives. Immunol. Lett..

[120] Dinkins C., Arko-Mensah J., Deretic V. (2010). Autophagy and HIV. Semin. Cell Dev. Biol..

[121] Kyei G.B., Dinkins C., Davis A.S., Roberts E., Singh S.B., Dong C., Wu L., Kominami E., Ueno T., Yamamoto A. (2009). Autophagy pathway intersects with HIV-1 biosynthesis and regulates viral yields in macrophages. J. Cell Biol..

[122] Gannagé M., Dormann D., Albrecht R., Dengjel J., Torossi T., Rämer P.C., Lee M., Strowig T., Arrey F., Conenello G. (2009). Matrix protein 2 of influenza A virus blocks autophagosome fusion with lysosomes. Cell Host Microbe.

[123] Orvedahl A., Alexander D., Tallóczy Z., Sun Q., Wei Y., Zhang W., Burns D., Leib D.A., Levine B. (2007). HSV-1 ICP34.5 confers neurovirulence by targeting the Beclin 1 autophagy protein. Cell Host Microbe.

[124] Stumptner-Cuvelette P., Jouve M., Helft J., Dugast M., Glouzman A.-S., Jooss K., Raposo G., Benaroch P. (2003). Human immunodeficiency virus-1 Nef expression induces intracellular accumulation of multivesicular bodies and major histocompatibility complex class II complexes: Potential role of phosphatidylinositol 3-kinase. Mol. Biol. Cell.

[125] Sandrin V., Cosset F.L. (2006). Intracellular versus cell surface assembly of retroviral pseudotypes is determined by the cellular localization of the viral glycoprotein, its capacity to interact with Gag, and the expression of the Nef protein. J. Biol. Chem..

[126] Sanfridson A., Hester S., Doyle C. (1997). Nef proteins encoded by human and simian immunodeficiency viruses induce the accumulation of endosomes and lysosomes in human T cells. Proc. Natl. Acad. Sci. USA.

[127] Arshad O., Gadawska I., Sattha B., Côté H.C.F., Hsieh A.Y.Y. (2018). Elevated cell-free mitochondrial DNA in filtered plasma is associated with HIV infection and inflammation. J. Acquir. Immune Defic. Syndr..

[128] Dai Z., Cai W., Hu F., Lan Y., Li L., Chung C., Caughey B., Zhang K., Tang X. (2015). Plasma mitochondrial DNA levels as a biomarker of lipodystrophy among HIV-infected patients treated with highly active antiretroviral therapy (HAART). Curr. Mol. Med..

[129] Younes S.-A., Talla A., Pereira Ribeiro S., Saidakova E.V., Korolevskaya L.B., Shmagel K.V., Shive C.L., Freeman M.L., Panigrahi S., Zweig S. (2018). Cycling CD4+ T cells in HIV-infected immune nonresponders have mitochondrial dysfunction. J. Clin. Investig..

[130] Brunet-Ratnasingham E., Dubé M., Kaufmann D.E. (2019). Targeting mitochondria to revive dysfunctional regulatory T cells. Trends Mol. Med..

[131] Heil M., Vega-Muñoz I. (2019). Nucleic acid sensing in mammals and plants: Facts and caveats. Int. Rev. Cell Mol. Biol..

[132] Hou Y., Dan X., Babbar M., Wei Y., Hasselbalch S.G., Croteau D.L., Bohr V.A. (2019). Ageing as a risk factor for neurodegenerative disease. Nat. Rev. Neurol..

[133] Cho B., Kim T., Huh Y.J., Lee J., Lee Y. (2019). Il Amelioration of mitochondrial quality control and proteostasis by natural compounds in Parkinson’s disease models. Int. J. Mol. Sci..

[134] White A.J., Wijeyekoon R.S., Scott K.M., Gunawardana N.P., Hayat S., Solim I.H., McMahon H.T., Barker R.A., Williams-Gray C.H. (2018). The peripheral inflammatory response to alpha-synuclein and endotoxin in Parkinson’s disease. Front. Neurol..

[135] Sliter D.A., Martinez J., Hao L., Chen X., Sun N., Fischer T.D., Burman J.L., Li Y., Zhang Z., Narendra D.P. (2018). Parkin and PINK1 mitigate STING-induced inflammation. Nature.

[136] Guerra F., Girolimetti G., Beli R., Mitruccio M., Pacelli C., Ferretta A., Gasparre G., Cocco T., Bucci C. (2019). Synergistic effect of mitochondrial and lysosomal dysfunction in Parkinson’s disease. Cells.

[137] Vilariño-Güell C., Wider C., Ross O.A., Dachsel J.C., Kachergus J.M., Lincoln S.J., Soto-Ortolaza A.I., Cobb S.A., Wilhoite G.J., Bacon J.A. (2011). VPS35 mutations in Parkinson disease. Am. J. Hum. Genet..

[138] Wang W., Wang X., Fujioka H., Hoppel C., Whone A.L., Caldwell M.A., Cullen P.J., Liu J., Zhu X. (2016). Parkinson’s disease-associated mutant VPS35 causes mitochondrial dysfunction by recycling DLP1 complexes. Nat. Med..

[139] Braschi E., Goyon V., Zunino R., Mohanty A., Xu L., McBride H.M. (2010). Vps35 mediates vesicle transport between the mitochondria and peroxisomes. Curr. Biol..

[140] Song P., Trajkovic K., Tsunemi T., Krainc D. (2016). Parkin modulates endosomal organization and function of the endo-lysosomal pathway. J. Neurosci..

[141] Small S.A., Petsko G.A. (2015). Retromer in Alzheimer disease, Parkinson disease and other neurological disorders. Nat. Rev. Neurosci..

[142] Nixon R.A. (2013). The role of autophagy in neurodegenerative disease. Nat. Med..

[143] Perrett R.M., Alexopoulou Z., Tofaris G.K. (2015). The endosomal pathway in Parkinson’s disease. Mol. Cell. Neurosci..

[144] Restelli L.M., Oettinghaus B., Halliday M., Agca C., Licci M., Sironi L., Savoia C., Hench J., Tolnay M., Neutzner A. (2018). Neuronal mitochondrial dysfunction activates the integrated stress response to induce fibroblast growth factor 21. Cell Rep..

[145] Deture M.A., Dickson D.W. (2019). The neuropathological diagnosis of Alzheimer’s disease. Mol. Neurodegener..

[146] D’Andrea M.R., Nagele R.G., Wang H.Y., Peterson P.A., Lee D.H.S. (2001). Evidence that neurones accumulating amyloid can undergo lysis to form amyloid plaques in Alzheimer’s disease. Histopathology.

[147] Willén K., Edgar J.R., Hasegawa T., Tanaka N., Futter C.E., Gouras G.K. (2017). Aβ accumulation causes MVB enlargement and is modelled by dominant negative VPS4A. Mol. Neurodegener..

[148] Wilquet V., Strooper B. (2004). De Amyloid-beta precursor protein processing in neurodegeneration. Curr. Opin. Neurobiol..

[149] Tang B.L. (2009). Neuronal protein trafficking associated with Alzheimer disease: From APP and BACE1 to glutamate receptors. Cell Adhes. Migr..

[150] Zhang Q.Y., Tan M.S., Yu J.T., Tan L. (2016). The role of retromer in Alzheimer’s disease. Mol. Neurobiol..

[151] Zafar S., Younas N., Correia S., Shafiq M., Tahir W., Schmitz M., Ferrer I., Andréoletti O., Zerr I. (2017). Strain-specific altered regulatory response of Rab7a and Tau in Creutzfeldt-Jakob disease and Alzheimer’s disease. Mol. Neurobiol..

[152] Rodriguez L., Mohamed N.V., Desjardins A., Lippé R., Fon E.A., Leclerc N. (2017). Rab7A regulates tau secretion. J. Neurochem..

[153] Kerr J.S., Adriaanse B.A., Greig N.H., Mattson M.P., Cader M.Z., Bohr V.A., Fang E.F. (2017). Mitophagy and Alzheimer’s Disease: Cellular and Molecular Mechanisms. Trends Neurosci..

[154] Fang E.F., Hou Y., Palikaras K., Adriaanse B.A., Kerr J.S., Yang B., Lautrup S., Hasan-Olive M.M., Caponio D., Dan X. (2019). Mitophagy inhibits amyloid-β and tau pathology and reverses cognitive deficits in models of Alzheimer’s disease. Nat. Neurosci..

[155] Du F., Yu Q., Yan S., Hu G., Lue L.F., Walker D.G., Wu L., Yan S.F., Tieu K., Yan S.S. (2017). PINK1 signalling rescues amyloid pathology and mitochondrial dysfunction in Alzheimer’s disease. Brain.

[156] Sorrentino V., Romani M., Mouchiroud L., Beck J.S., Zhang H., D’Amico D., Moullan N., Potenza F., Schmid A.W., Rietsch S. (2017). Enhancing mitochondrial proteostasis reduces amyloid-β proteotoxicity. Nature.

[157] Melber A., Haynes C.M. (2018). UPRmt regulation and output: A stress response mediated by mitochondrial-nuclear communication. Cell Res..

[158] Wang Z.T., Lu M.H., Zhang Y., Ji W.L., Lei L., Wang W., Fang L.P., Wang L.W., Yu F., Wang J. (2019). Disrupted-in-schizophrenia-1 protects synaptic plasticity in a transgenic mouse model of Alzheimer’s disease as a mitophagy receptor. Aging Cell.

[159] Cummins N., Tweedie A., Zuryn S., Bertran-Gonzalez J., Götz J. (2019). Disease-associated tau impairs mitophagy by inhibiting Parkin translocation to mitochondria. EMBO J..

[160] Fang E.F. (2019). Mitophagy and NAD^+^ inhibit Alzheimer disease. Autophagy.

[161] Heppner F.L., Ransohoff R.M., Becher B. (2015). Immune attack: The role of inflammation in Alzheimer disease. Nat. Rev. Neurosci..

[162] Wang W.Y., Tan M.S., Yu J.T., Tan L. (2015). Role of pro-inflammatory cytokines released from microglia in Alzheimer’s disease. Ann. Transl. Med..

[163] Holmes C., Butchart J. (2011). Systemic inflammation and Alzheimer’s disease. Biochem. Soc. Trans..

[164] Dutta D., Calvani R., Bernabei R., Leeuwenburgh C., Marzetti E. (2012). Contribution of impaired mitochondrial autophagy to cardiac aging: Mechanisms and therapeutic opportunities. Circ. Res..

[165] Marzetti E., Wohlgemuth S.E., Anton S.D., Bernabei R., Carter C.S., Leeuwenburgh C. (2009). Cellular mechanisms of cardioprotection by calorie restriction: State of the science and future perspectives. Clin. Geriatr. Med..

[166] Sun N., Yun J., Liu J., Malide D., Liu C., Rovira I.I., Holmström K.M., Fergusson M.M., Yoo Y.H., Combs C.A. (2015). Measuring in vivo mitophagy. Mol. Cell.

[167] Egan D., Kim J., Shaw R.J., Guan K.-L. (2011). The autophagy initiating kinase ULK1 is regulated via opposing phosphorylation by AMPK and mTOR. Autophagy.

[168] Maejima Y., Isobe M., Sadoshima J. (2016). Regulation of autophagy by Beclin 1 in the heart. J. Mol. Cell. Cardiol..

[169] Tan V.P., Miyamoto S. (2016). Nutrient-sensing mTORC1: Integration of metabolic and autophagic signals. J. Mol. Cell. Cardiol..

[170] Ock S., Lee W.S., Ahn J., Kim H.M., Kang H., Kim H.S., Jo D., Abel E.D., Lee T.J., Kim J. (2016). Deletion of IGF-1 receptors in cardiomyocytes attenuates cardiac aging in male mice. Endocrinology.

[171] Sciarretta S., Zhai P., Shao D., Maejima Y., Robbins J., Volpe M., Condorelli G., Sadoshima J. (2012). Rheb is a critical regulator of autophagy during myocardial ischemia: Pathophysiological implications in obesity and metabolic syndrome. Circulation.

[172] Fan H., He Z., Huang H., Zhuang H., Liu H., Liu X., Yang S., He P., Yang H., Feng D. (2020). Mitochondrial quality control in cardiomyocytes: A critical role in the progression of cardiovascular diseases. Front. Physiol..

[173] Tong M., Saito T., Zhai P., Oka S.I., Mizushima W., Nakamura M., Ikeda S., Shirakabe A., Sadoshima J. (2019). Mitophagy Is essential for maintaining cardiac function during high fat diet-induced diabetic cardiomyopathy. Circ. Res..

[174] Shang X., Lin K., Zhang Y., Li M., Xu J., Chen K., Zhu P., Yu R. (2020). Mst1 deletion reduces septic cardiomyopathy via activating Parkin-related mitophagy. J. Cell. Physiol..

[175] Hoshino A., Wang W., Wada S., McDermott-Roe C., Evans C.S., Gosis B., Morley M.P., Rathi K.S., Li J., Li K. (2019). The ADP/ATP translocase drives mitophagy independent of nucleotide exchange. Nature.

[176] Wu S., Lu Q., Wang Q., Ding Y., Ma Z., Mao X., Huang K., Xie Z., Zou M.H. (2017). Binding of FUN14 Domain Containing 1 with Inositol 1,4,5-Trisphosphate Receptor in Mitochondria-Associated endoplasmic reticulum membranes maintains mitochondrial dynamics and function in hearts in vivo. Circulation.

[177] Yu W., Xu M., Zhang T., Zhang Q., Zou C. (2019). Mst1 promotes cardiac ischemia–reperfusion injury by inhibiting the ERK-CREB pathway and repressing FUNDC1-mediated mitophagy. J. Physiol. Sci..

[178] Quinsay M.N., Thomas R.L., Lee Y., Gustafsson Å.B. (2010). Bnip3-mediated mitochondrial autophagy is independent of the mitochondrial permeability transition pore. Autophagy.

[179] Liu L., Sakakibara K., Chen Q., Okamoto K. (2014). Receptor-mediated mitophagy in yeast and mammalian systems. Cell Res..

[180] Chaanine A.H., Jeong D., Liang L., Chemaly E.R., Fish K., Gordon R.E., Hajjar R.J. (2012). JNK modulates FOXO3a for the expression of the mitochondrial death and mitophagy marker BNIP3 in pathological hypertrophy and in heart failure. Cell Death Dis..

[181] Smyrnias I., Gray S.P., Okonko D.O., Sawyer G., Zoccarato A., Catibog N., López B., González A., Ravassa S., Díez J. (2019). Cardioprotective effect of the mitochondrial unfolded protein response during chronic pressure overload. J. Am. Coll. Cardiol..

[182] Gong D., Zhang H., Hu S. (2013). Mitochondrial aldehyde dehydrogenase 2 activation and cardioprotection. J. Mol. Cell. Cardiol..

[183] Wu B., Yu L., Wang Y., Wang H., Li C., Yin Y., Yang J., Wang Z., Zheng Q., Ma H. (2016). Aldehyde dehydrogenase 2 activation in aged heart improves the autophagy by reducing the carbonyl modification on SIRT1. Oncotarget.

[184] Cadete V.J.J., Deschênes S., Cuillerier A., Brisebois F., Sugiura A., Vincent A., Turnbull D., Picard M., McBride H.M., Burelle Y. (2016). Formation of mitochondrial-derived vesicles is an active and physiologically relevant mitochondrial quality control process in the cardiac system. J. Physiol..

[185] Servier Medical Art. http://www.servier.com/Powerpoint-image-bank.

